# Treatment Efficacy of Electromyography versus Fiberscopy-Guided Botulinum Toxin Injection in Adductor Spasmodic Dysphonia Patients: A Prospective Comparative Study

**DOI:** 10.1155/2014/327928

**Published:** 2014-10-14

**Authors:** Jae Wook Kim, Jae Hong Park, Ki Nam Park, Seung Won Lee

**Affiliations:** Department of Otolaryngology—Head and Neck Surgery, Soonchunhyang University College of Medicine, 1174 Jung-Dong, Bucheon 420-021, Republic of Korea

## Abstract

*Introduction*. This study prospectively evaluates and compares the treatment efficacy of botulinum toxin injection under electromyography guidance (EMG group) and percutaneous botulinum toxin injection under flexible fiberscopic guidance (fiberscopy group).* Methods*. Thirty patients with adductor spasmodic dysphonia (ADSD), who had never received treatment, were randomly allocated into EMG- or fiberscopy-guided botulinum toxin injections between March 2008 and February 2010. We assessed acoustic and aerodynamic voice parameters, and the voice handicap index (VHI) before injection and at 1, 3, and 6 months after injection. *Results*. The mean total dosage of botulinum toxin was similar for both groups: 1.7 ± 0.5 U for the EMG group and 1.8 ± 0.4 U for the fiberscopy group (*P* > 0.05). There were no significant differences in outcomes between the two groups in either the duration of effectiveness or complications such as breathy voice and aspiration.* Conclusion*. Botulinum toxin injection under fiberscopic guidance is a viable alternative to EMG-guided botulinum toxin injection for the treatment of adductor spasmodic dysphonia when EMG equipment is unavailable.

## 1. Introduction

Botulinum toxin injection is widely accepted as an effective treatment modality for controlling the symptoms of adductor spasmodic dysphonia (ADSD) [1]. There are a variety of injection approaches to deliver botulinum toxin to the thyroarytenoid (TA) muscle, including EMG guidance, the point-touch technique, a transnasal or transoral approach, and percutaneous fiberscopic guidance [2–5]. However, no studies have compared the treatment efficacy of the various techniques.

Although botulinum toxin injection under EMG guidance is the standard technique for delivering botulinum toxin to the TA muscle, not all otolaryngology departments have EMG equipment, due to its high cost. When EMG equipment is not available, fiberscopic guidance could be an alternative. However, the selection of injection technique may ultimately be determined by equipment availability, surgeon preference, and/or training, without reference of clinical evidence.

The purpose of this prospective study was to determine the treatment efficacy of EMG guided and fiberscopy-guided botulinum toxin injection in the treatment of ADSD patients.

## 2. Materials and Methods

### 2.1. Patients

Thirty untreated adductor spasmodic dysphonia (ADSD) patients were randomly enrolled in this prospective study between March 2008 and February 2010 at the Department of Otolaryngology—Head and Neck surgery, College of Medicine, Soonchunhyang University in Bucheon, Republic of Korea. All patients were untreated, and we excluded patients who previously received any treatment for ADSD including medication, botulinum toxin injections, selective recurrent laryngeal nerve section, and speech therapy.

The study population included 15 patients in the EMG group and 15 in the fiberscopy group. The study design was approved by our Institutional Review Board prior to the start of the study (SCHBC IRB 09 10).

### 2.2. Botulinum Toxin Injection Treatment Protocol

#### 2.2.1. EMG Group

The patient was placed in a supine position with neck extension and a 26-gauge Teflon-coated injection needle connected to a 1-cc syringe preloaded with botulinum toxin (BOTOX, Allergan Inc, Irvine, CA, USA) was inserted through the cricothyroid membrane into the TA muscle under EMG guidance. The needle was adjusted, while the patient was holding their breath until crisp motor unit action potentials were elicited; botulinum toxin was then injected.

#### 2.2.2. Fiberscopy Group

The patient was placed in a semisitting position with neck extension. Before the procedure, a 4% lidocaine spray was applied onto the nasal cavity, pharynx, and larynx. The botulinum toxin was injected through the cricothyroid membrane, directly into the vocalis muscle, using a disposable 25-G long needle under transnasal flexible fiberscopic monitoring (Olympus ENF type V2 Rhino Laryngo Videoscope, Olympus Medical System, Tokyo, Japan). The appropriate needle location was confirmed through the flexible fiberscope prior to injection [6].

All botulinum toxin injections in both the EMG and the fiberscopy group were performed by a single experienced laryngologist (SW Lee).

### 2.3. Voice Parameter Evaluation

Acoustic and aerodynamic analyses were conducted before treatment and at 1, 3, and 6 months after botulinum toxin injection by a single speech language pathologist. The percentages of jitter, shimmer, and data for the harmonics-to-noise ratio (HNR) were collected using the Multidimensional Voice Program (MDVP model 4500; Kay Pentax, Lincoln Park, NJ, USA). Maximum phonation time (MPT) data were collected using the computerized speech lab (CSL model 4500; Kay Pentax, NJ, USA). Psychosocial data were collected using the Korean language version of the voice handicap index (VHI-30). A 10-point visual analog scale (VAS) was used to measure patient satisfaction with the injection procedure itself (0 = worst, 5 = fair, and 10 = best). Following the injection, patients kept daily diaries to record side effects such as breathiness and aspiration.

### 2.4. Statistical Analysis

Statistical analyses were performed using the Wilcoxon signed-rank test and the Mann-Whitney test (Korean Version of SPSS 17.0 for Windows). *P* values less than 0.05 were considered to indicate statistical significance.

## 3. Results

### 3.1. Treatment Efficacy and Side Effects

The mean dosage of botulinum toxin was similar in both groups, 1.7 ± 0.5 U for the EMG group and 1.8 ± 0.4 U for the fiberscopy group. Total dosages were not significantly different between the two groups (*P* > 0.05).

Table 1 presents patient demographic data and a summary of results for both groups. The mean effective duration was 3.7 ± 0.8 months for the EMG group and 4.7 ± 1.4 months for the fiberscopy group. The difference in mean effective duration between the groups was not statistically significant (*P* > 0.05).

The mean patient satisfaction score for the procedure as measured by VAS was 6.4 ± 2.4 for the fiberscopy group and 7.7 ± 1.5 for the EMG group. Patient satisfaction scores were significantly more favorable in the EMG group compared to the fiberscopy group (*P* < 0.05). The mean duration of breathiness following injection was 8.7 ± 5.5 days for the EMG group and 10.7 ± 8.3 days for the fiberscopy group. The mean duration of aspiration was 7.1 ± 4.7 days in the EMG group and 8.9 ± 7.5 days in the fiberscopy group. No significant differences were identified in breathiness or aspiration period following injection (*P* > 0.05).

### 3.2. Voice Parameters

Tables 2 and 3 show the voice parameter analysis of the EMG and fiberscopy groups, respectively. The average MPT, jitter, shimmer, harmonics-to-noise ratio (HNR), and fundamental frequency did not change significantly following botulinum toxin injection in either the EMG or fiberscopy group.

However, the mean VHI value was significantly improved following injection in the EMG group from 81.9 ± 19.7 to 23.3 ± 6.3 (*P* = 0.002). The mean VHI value was also significantly improved in the fiberscopy group following injection from 77.5 ± 24.3 to 23.4 ± 5.2 (*P* = 0.001).

There were no statistically significant differences in voice parameters between the EMG and fiberscopy groups (*P* > 0.05).

## 4. Discussion

Blitzer et al. were the first to inject botulinum toxin into the vocal folds to treat ADSD in 1984 [7]. Since that time, botulinum toxin injection under EMG guidance, has been established as the “gold standard” in ADSD treatment [2, 8].

A variety of techniques have been developed to deliver botulinum toxin to the vocal folds, such as a transnasal approach, a transoral approach, percutaneous fiberscopic guidance, and EMG guidance [4, 9]. However, no studies have compared the efficacy of these techniques.

The advantage of EMG guidance is the confirmation of needle's placement within the TA muscle by showing distinct motor unit action potential (MUAP) with phonation [8]. However, to confirm the correct needle location within the TA muscle, the operator must be familiar with EMG and possess the technical knowledge to interpret EMG signals. Moreover, a department must purchase an EMG machine to use this technique, which can be cost prohibitive.

The point-touch technique, an alternative to EMG guidance that relies on anatomical landmarks can be performed rapidly and be well tolerated by the patient. But this technique is a true blind technique and visualization of the vocal folds may be required to confirm accurate placement of the needle tip in the TA muscle. Therefore, substantial experience and technical expertise are mandatory [10].

Fiberscopy-guided percutaneous injection has the advantages of both EMG guidance and the point-touch technique. It can be performed without an EMG machine, it can visualize the vocal folds during the procedure, and it demonstrates high reliability due to its ability to confirm needle location. In addition, this technique is a modification of injection laryngoplasty techniques that are familiar to laryngologists. However, the need for local anesthetic spray for pharynx to aid in the insertion of the fiberscope could also be a source of discomfort.

In this study, the only significant difference between the EMG and fiberscopy groups was the patient satisfaction score with the procedure. The fiberscopy group had a significantly lower satisfaction score than the EMG group (6.4 ± 2.4 versus 7.7 ± 1.5). This could have been due to the discomfort experience related with the fiberscopy procedure.

This study is the first randomized clinical trial to compare the efficacy of EMG- and fiberscopy-guided botulinum toxin injections for the treatment of ADSD in untreated patients. However, the small study population (15 patients per group) could be the limitation.

Based upon our acoustic, aerodynamic, and VHI results, percutaneous botulinum toxin injection under fiberscopic guidance is as effective as that under EMG guidance. When an EMG machine is not available, percutaneous botulinum toxin injection under fiberscopic guidance is a viable alternative for treating ADSD patients. We suggest that the optimum injection technique be determined by the surgeon's training, equipment availability, and preferences.

In conclusion, percutaneous botulinum toxin injection under fiberscopic guidance is as effective as under EMG guidance and could be a viable alternative for botulinum toxin injection in the treatment of adductor spasmodic dysphonia.

## Figures and Tables

**Table 1:** Patient demographics and results of EMG and fiberscopy-guided injections.

	EMG group	Fiberscopy group	*P* value
Mean ages (years)	34.9 ± 14.9	31.9 ± 9.7	0.863
Total dosage of botulinum toxin (U)	1.7 ± 0.5	1.8 ± 0.4	0.814
Duration breathiness (days)	8.7 ± 5.5	10.7 ± 8.3	0.584
Duration aspiration (days)	7.1 ± 4.7	8.9 ± 7.5	0.626
Mean effective duration (months)	3.7 ± 0.8	4.7 ± 1.4	0.150
VAS patient satisfaction score	7.7 ± 1.5	6.4 ± 2.4	0.048∗

∗Significant differences of patient satisfaction scores using the visual analog scales (VAS).

**Table 2:** Voice analysis of EMG group following botulinum toxin injection.

	Pre-botox	Post-botox	*P* value
MPT (sec)	12.11 ± 1.78	12.14 ± 1.90	0.776
Jitter (%)	1.66 ± 1.15	1.21 ± 0.45	0.233
Shimmer (%)	4.63 ± 2.10	3.40 ± 1.29	0.140
HNR (dB)	21.89 ± 3.95	23.35 ± 6.36	0.300
*F*_0_ (dB)	209.2 ± 28.9	193.65 ± 20.9	0.173
VHI	81.9 ± 19.7	23.3 ± 6.3	0.002∗

Pre-botox: before botulinum toxin injection.

Post-botox: after botulinum toxin injection.

∗Significantly improved after botulinum toxin injection. Analyzed by Wilcoxon signed-rank test.

MPT: maximum phonation time.

HNR: harmonics-to-noise ratio.

*F*_0_ (dB): fundamental frequency (decibel).

VHI: voice handicap index-30.

**Table 3:** Voice analysis of fiberscopy group following botulinum toxin injection.

	Pre-botox	Post-botox	*P* value
MPT (sec)	11.1 ± 2.7	11.3 ± 1.7	0.691
Jitter (%)	1.73 ± 1.58	1.07 ± 1.11	0.256
Shimmer (%)	4.11 ± 2.69	2.89 ± 1.07	0.177
HNR (dB)	21.98 ± 7.2	23.4 ± 5.2	0.532
*F*_0_ (dB)	218.1 ± 38.8	205.5 ± 27.9	0.460
VHI	77.5 ± 24.3	23.4 ± 5.2	0.001∗

Pre-botox: before botulinum toxin injection.

Post-botox: after botulinum toxin injection.

∗Significantly improved after botulinum toxin injection. Analyzed by Wilcoxon signed-rank test.

MPT: maximum phonation time.

HNR: harmonics-to-noise ratio.

*F*_0_ (dB): fundamental frequency (decibel).

VHI: voice handicap index-30.
